# Supplementary material for: Study on the Printability through Digital Light Processing Technique of Ionic Liquids for CO2 Capture
Source: Polymers (Basel). 2019 Nov 23;11(12):1932. doi: 10.3390/polym11121932 (PMC6960677; doi:10.3390/polym11121932)
Supplement: Supplementary file 1 [file polymers-11-01932-s001.pdf]

Study on the printability through Digital Light Processing technique of ionic liquids for CO_2 capture

Matteo Gillono[1,2], Annalisa Chiappone[1], Lorenzo Mendola[1], Manuel Gomez Gomez[1], Luciano Scaltrito[1], Candido Fabrizio Pirri[1,2], Ignazio Roppolo[1]*

[1] Department of Applied Science and Technology, Politecnico di Torino, Corso Duca degli Abruzzi 24, 10129, Torino, Italy
[2] Center for Sustainable Future Technologies @Polito, Istituto Italiano di Tecnologia, Via Livorno, 60, 10144, Torino, Italy
* Correspondence: ignazio.roppolo@polito.it; Tel.: +39-0110907412

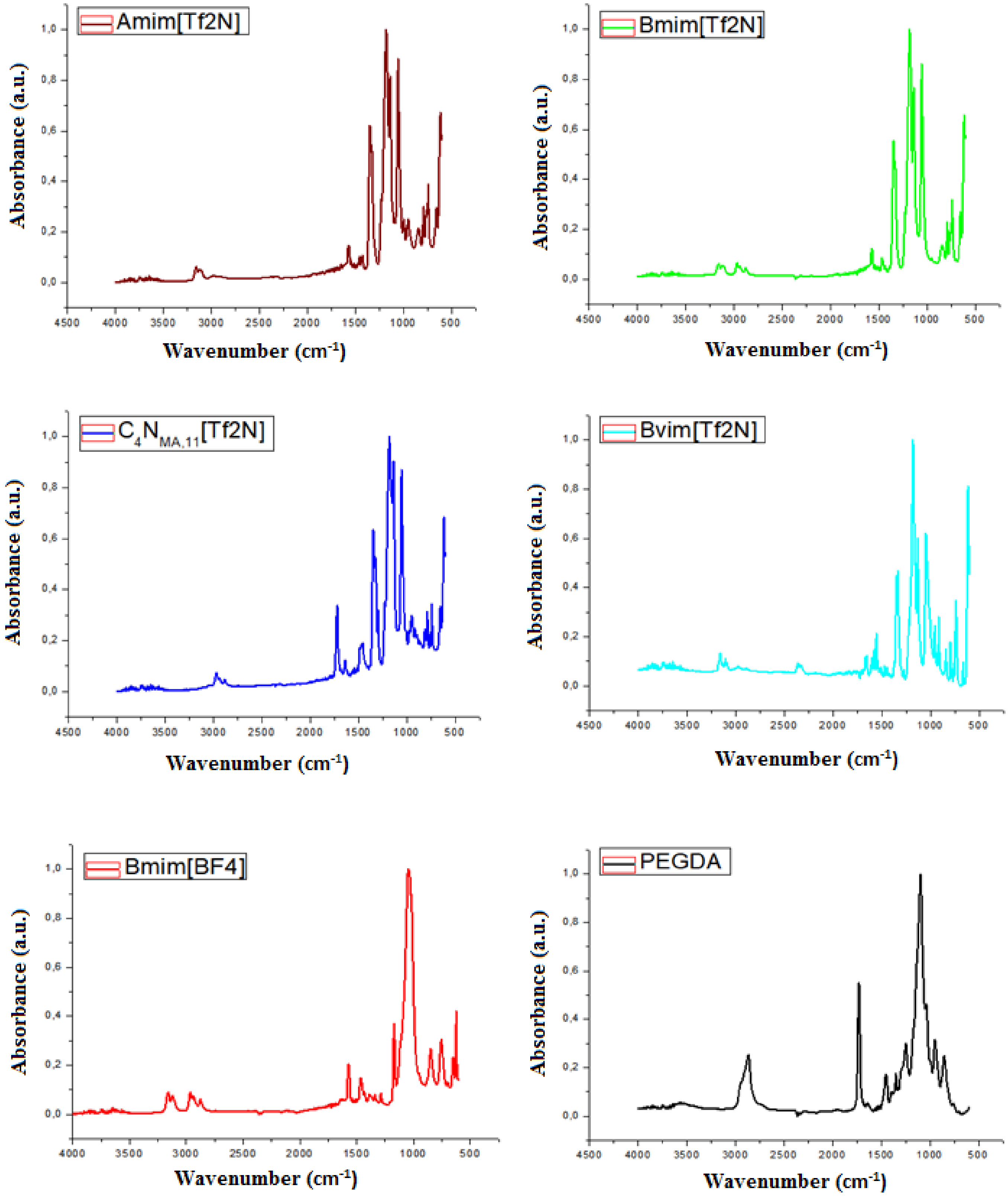

Figure S1 FTIR-ATR spectra of neat ILs and neat PEGDA.

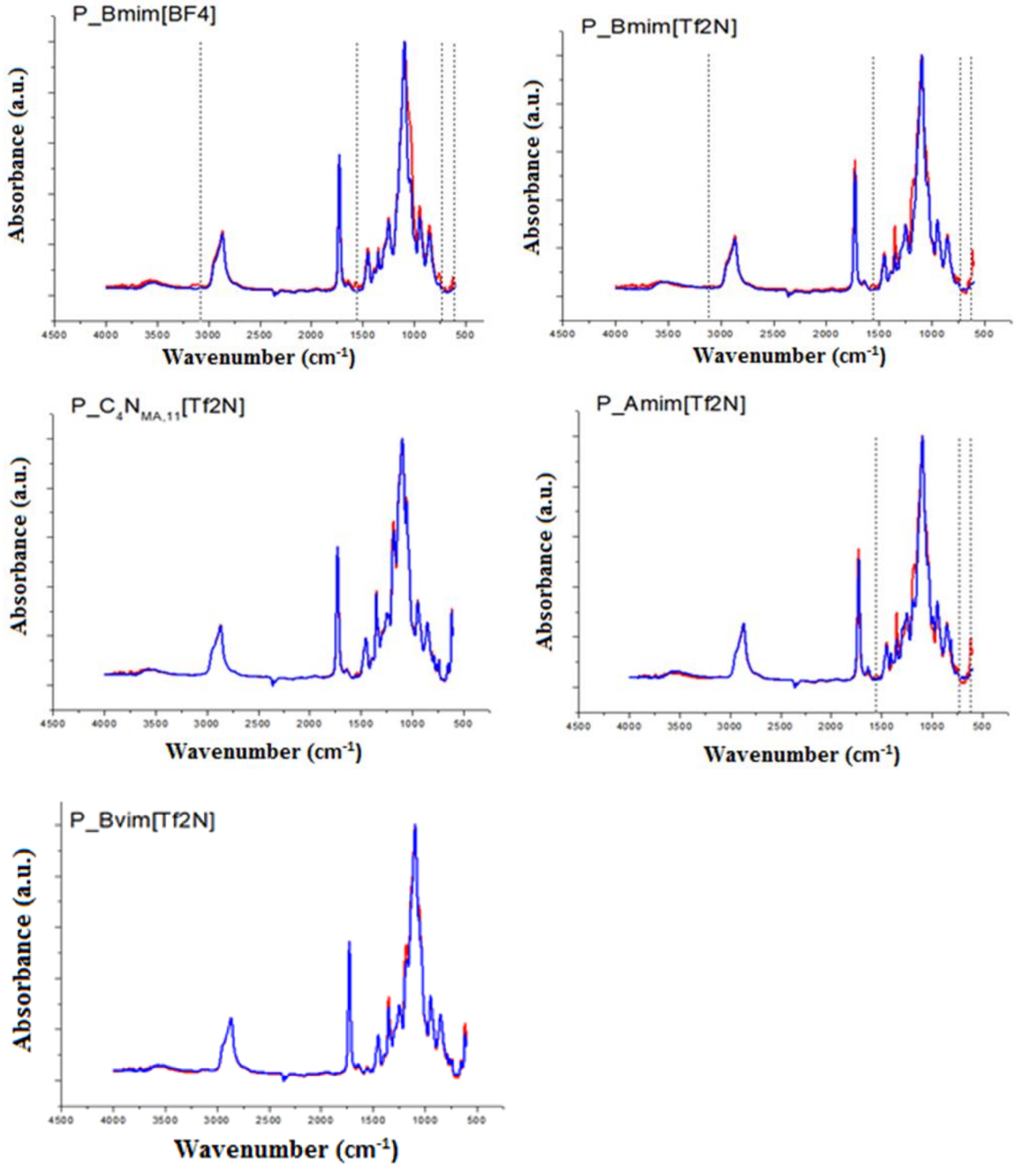

Figure S2 FTIR-ATR spectra of polymerized formulations before (red) and after (blue) solvent washing.

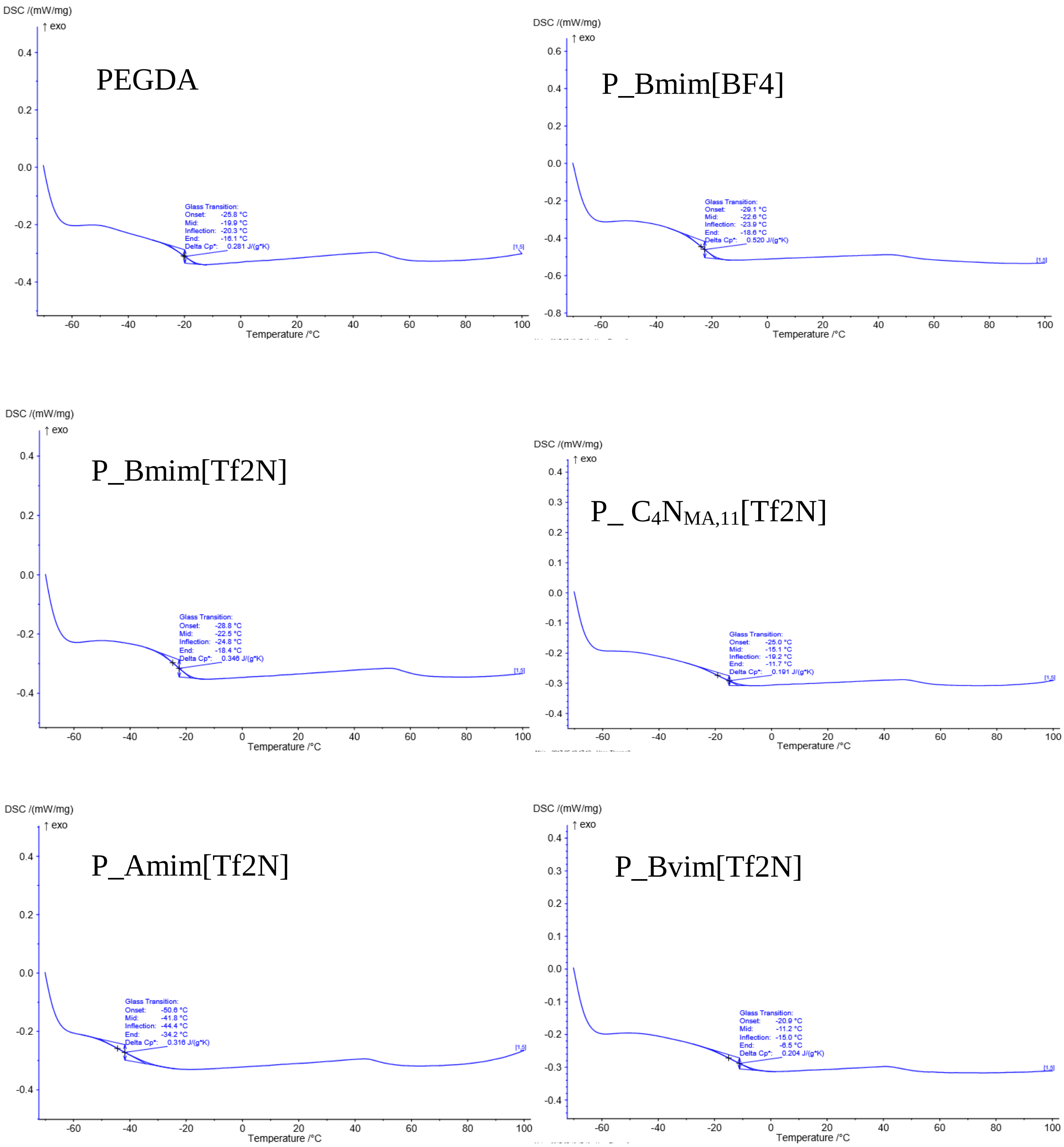

Figure S3 DSC analysis of the studied formulations.

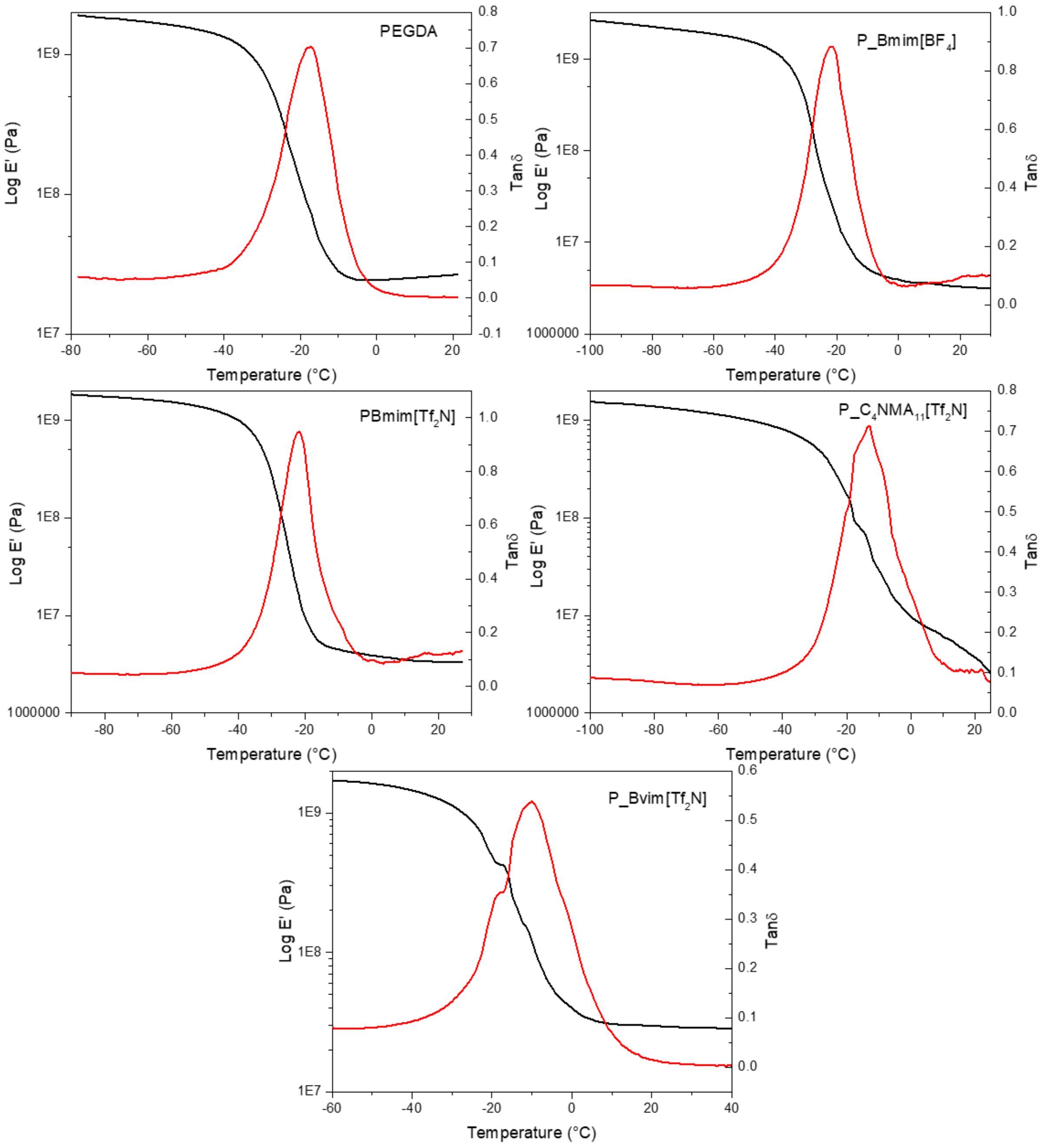

Figure S4 DMTA analysis on the polymerized samples.

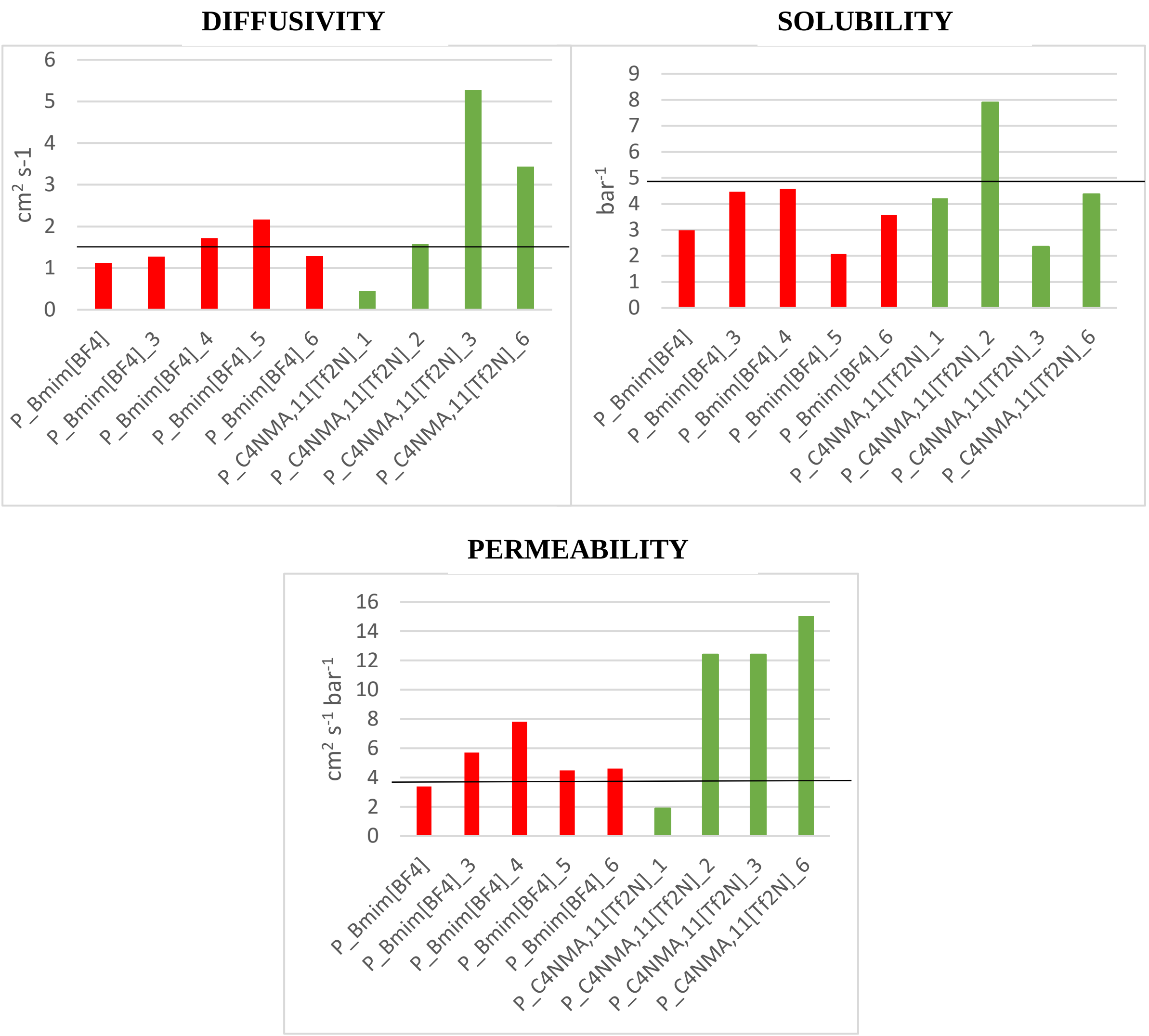

Figure S5 Diffusivity, solubility and permeability values of membranes containing increasing concentrations Bmim[BF4](red) e $C_4N_{MA,11}$[Tf2N] (green). The black line indicates the value calculated for neat PEGDA.
